# Microbial Interventions in Bioremediation of Heavy Metal Contaminants in Agroecosystem

**DOI:** 10.3389/fmicb.2022.824084

**Published:** 2022-05-06

**Authors:** Veni Pande, Satish Chandra Pandey, Diksha Sati, Pankaj Bhatt, Mukesh Samant

**Affiliations:** ^1^Cell and Molecular Biology Laboratory, Department of Zoology (DST-FIST Sponsored), Soban Singh Jeena University Campus, Almora, India; ^2^Department of Biotechnology, Sir J C Bose Technical Campus, Kumaun University, Bhimtal, India; ^3^Department of Zoology, Kumaun University, Nainital, India; ^4^Department of Agricultural and Biological Engineering, PurdueUniversity, West Lafayette, IN, United States

**Keywords:** heavy metals, bioremediation, biosorption, biotransformation, bioleaching

## Abstract

Soil naturally comprises heavy metals but due to the rapid industrialization and anthropogenic events such as uncontrolled use of agrochemicals their concentration is heightened up to a large extent across the world. Heavy metals are non-biodegradable and persistent in nature thereby disrupting the environment and causing huge health threats to humans. Exploiting microorganisms for the removal of heavy metal is a promising approach to combat these adverse consequences. The microbial remediation is very crucial to prevent the leaching of heavy metal or mobilization into the ecosystem, as well as to make heavy metal extraction simpler. In this scenario, technological breakthroughs in microbes-based heavy metals have pushed bioremediation as a promising alternative to standard approaches. So, to counteract the deleterious effects of these toxic metals, some microorganisms have evolved different mechanisms of detoxification. This review aims to scrutinize the routes that are responsible for the heavy metal(loid)s contamination of agricultural land, provides a vital assessment of microorganism bioremediation capability. We have summarized various processes of heavy metal bioremediation, such as biosorption, bioleaching, biomineralization, biotransformation, and intracellular accumulation, as well as the use of genetically modified microbes and immobilized microbial cells for heavy metal removal.

## Introduction

**FIGURE 1:**
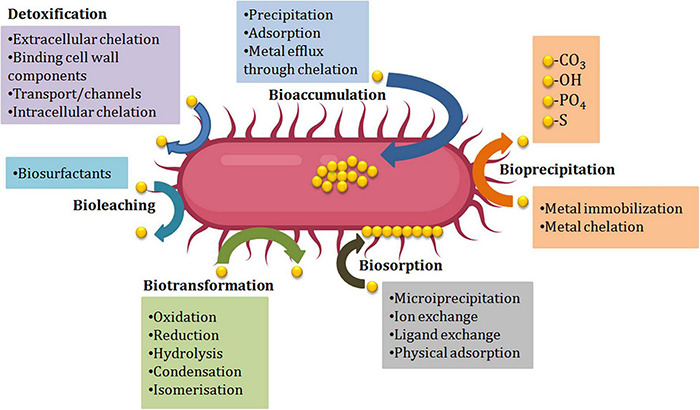
The primary sources and effects of heavy metal exposure at various trophic levels.

Contamination of heavy metals (HMs) has widely spread all over the world and therefore is a primary matter of concern as it poses threat to animals, plants as well as humans and disturbs the environment. HMs, like other metals and metalloids, are present in the earth’s crust, however, the recalcitrant nature of HMs makes them resistant to degradation. Bioaccumulation of HMs and metalloids via different sources like air, water, causes them to infiltrate plants, animals, and humans, as well as the advancement of the food chain over time (Briffa et al., 2020). Several natural and man-made processes could release these HMs into the environment (Dembitsky and Rezanka, 2003). Due to the growing usage of agrochemicals and inorganic fertilizers, modern agricultural methods have resulted in agricultural pollution, resulting in the ecosystem and environmental destruction (Malik et al., 2017). HMs are also introduced into agricultural systems through the use of sewage sludge and organic waste manure, industrial wastes, and wastewater irrigation (Srivastava et al., 2016; Sharma et al., 2017) (Figure 1). Extraction of HMs from their ores occurs during the processing of minerals and throughout this process, some portions are left out in the open and get relocated to different places due to flood and wind thus causing serious environmental hazards. The essential nourishment of food crops is the soil and therefore agrarian soil is of huge concern owing to its linkage with the production of food, which could affect the health of living organisms. Despite being part of the soil, HMs cause serious harm to the soil as well as plants in their concentrated form. Thus, they are considered to be hazardous (Osmani et al., 2015). HMs are responsible for not only changing the composition of soil but also forming the basis of stress in the plants resulting in the failure of the crop. Biological molecules like lipids, nucleic acids, proteins, and enzymes get damaged due to the production of free radicals by the HMs thus increasing intracellularly the reactive oxygen species (ROS) levels thereby leading to oxidative stress. The failure in all of these biological substances creates several physiological issues, including, cell damage, DNA damage, and enzyme inhibition, all of which can lead to the plant’s death (Wu et al., 2016). HM pollution in modern agriculture has become a severe challenge in most emerging and underdeveloped countries due to a variety of social-economical, scientific, and developmental difficulties. Discovering environmentally safe, long-term solutions to the HM contamination problem is a serious task. Currently, the use of microorganisms or functional biocatalysts in the remediation of soil contaminated with HMs entails the integration of genomes, transcriptomics, proteomics, signaling systems, and synthetic biology knowledge (Hemmat-Jou et al., 2018). These strategies offer a new vista in biotechnology, allowing for the creation of a complex biological system to produce a better microbial system capable of combating HMs contamination (Sayqal and Ahmed, 2021). HMs affect the soil microbiology and modify rhizospheric connections between plants and microorganisms, influencing soil characteristics, plant growth, vegetation type, and agricultural land production, among other things. Different microbial communities with specific metabolic capacities reside in the soil, e.g., organic substances are formed by certain microorganisms while interacting with toxic metals whereas some other assists in the formation of natural nanoparticles thus reducing HMs (Wang and Chen, 2006). Owing to their high surface area to volume ratio, which is associated directly with their increased reactivity, nanotechnology-based materials have also been explored for HMs micro-remediation (Vijayaraj et al., 2019). The approaches and successes of biotechnological applications for environmental protection, decontamination, and the elimination of HMs and metalloids have thus been covered in this study.

The advancement of biotechnological applications and strategies for environmental protection, detoxification, and the removal of HMs and metalloids are the subject of this review. The goal of this review is to compile a list of key findings on HM contamination in modern agriculture, as well as to sketch a probable research roadmap for the future. The review explored the depth information about the mechanism and impacts of the HMs in microbial systems.

## Heavy Metal Pollution in Agroecosystem: Consequences and Plant Responses

### Effect on Soil Health, Fertility, and Microbial Dynamics

Soil biology plays a vital part in maintaining healthy soil quality, which is crucial for sustainable agriculture. The anthropogenic activities are central in contaminating soil with HMs, e.g., industrial, mining, and agricultural operations as the s present in mining waste, sewage sludge, inorganic fertilizers, and pesticides, tend to disturb soil microbes by percolating into the soil environment (Gupta et al., 2010; Tóth et al., 2016; Sharma et al., 2017). With rising amounts of HM pollution, microbial viability declines. Yuan et al. (2015) showed that microbial survivability was found to be negatively correlated with prolonged Pb exposure. According to de Quadros et al. (2016), coal mining operations; result in a decrease in microbial biomass, abundance, and variability. Nayak et al. (2015) reported that up to 40 and 100% fly ash amendments resulted in better microbial population dynamics with increased concentrations of Zn, Fe, Cu, Mn, Cd, and Cr in agricultural soils. Total microbial activity, as determined by the fluoresceindiacetate (FDA) test, and denitrifiers, on the contrary, exhibited an increasing tendency of up to 40% fly ash addition. The application of fly ash, on the other hand, reduced the activity of both acid and alkaline phosphatase. Various types of HM toxicity and their harmful effects on soil, plants, and humans are presented in Table 1.

**TABLE 1:** Various types of heavy metal toxicity and their harmful effects on soil, plants, and humans.

Heavy metals	Toxicity form	Soil	Plant	Health risks	References
Cd	Cd^2+^	Destroy microbes, take up organic material, and alter the physical and chemical properties of soil.	Decrease root length and biomass, prevent germination of seeds, and limits stem conductance.	Negatively affects renal function, hampers functioning of sex hormones, acts as an endocrine disruptor	Peralta-Videa et al., 2009; Jibril et al., 2017; Vardhan et al., 2019
Pb	Pb2+	Alter soil pH, change absorption ability of soil, and declining fertility.	Diminish chlorophyll content, reduce protein content, causes shortened leaves and cause damage to DNA.	Encephalopathy affects CNS, cardiovascular, and circulatory systems	El-Kady and Abdel-Wahhab, 2018; Cai et al., 2019; Lan et al., 2020
Cu	Cu salts	Urease activity loss, influence microbes dynamics, and lessen oxidation ability	Disturbs root growth, reduce shoot length and polypeptide, and shift lipid content.	Hampers normal metabolism and affect kidney functions	Hough et al., 2004; Caetano et al., 2016; Panagos et al., 2018
Zn	Zn^2+^	Modify soil pH, bicarbonate, and organic material level, and blocks enzyme function.	Deviation in enzyme function, element transport blockage, and interveinal chlorosis.	Respiratory problems	Hough et al., 2004; Plum et al., 2010

### Effect on Soil Microbial Functions and Processes

Due to HM toxicity, litter breakdown is slowed, resulting in an uneven litter deposit on the soil (Illmer and Schinner, 1991; Giller et al., 1998; Marschner and Kalbitz, 2003). Kozlov and Zvereva (2015) investigated the breakdown rate of mountain birch (*Betula pubescens* ssp. *czerepanovii*) leaves in a significantly contaminated industrial setting close to the nickel-copper smelter in Monchegorsk. During 2 years of exposure, there was a substantial reduction of 49% in the relative weight of native leaves compared to the loss observed in the unpolluted forest. Furthermore, anthropogenic HM contamination has been found in a number of studies to have a negative impact on stream litter decomposition (Carlisle and Clements, 2005; Hogsden and Harding, 2012; Ferreira et al., 2016). In both ecological toxicology and ecological tracking investigations, the rate of soil organic carbon mineralization has been routinely utilized as a test for metal toxicity (Giller et al., 1998). Carbon mineralization may be measured using the soil respiration rate. A negative association was found between soil microbial respiration and HM concentration by Nwuche and Ugoji (2008). From an average rate of 2.51–2.56 g of C/g at the start of the trial, the rate of soil microbial respiration was lowered to 0.98, 1.08, and 1.61 g of C/g in the Cu: Zn, Cu, and Zn treated soils, respectively. Because of differences in the experimental designs, fluctuations in soil characteristics, and substrate concentrations, HM exposure can either stimulate or impede N-mineralization. HM pollution disrupts nitrogen transformation pathways, which ultimately affects N-mineralization (Dai et al., 2004; Vásquez-Murrieta et al., 2006; Hamsa et al., 2017). HM pollution has a similar impact on both N mineralization as well as nitrification i.e., both processes tend to decrease with an increasing amount of HM pollutants (deCatanzaro and Hutchinson, 1985). Furthermore, nitrification is more susceptible to HM contamination than N mineralization (Rother et al., 1982; Bewley and Stotzky, 1983).

### Impact on Soil Enzymes

Metal composition, pH of the soil, organic matter, and clay content are important factors regulating the biological availability of metals in the soil. HMs affect soil enzymatic activity such as alkaline phosphatase, arylsulfatase, β-glucosidase, cellulase, dehydrogenase, invertase, protease, and urease (Oliveira and Pampulha, 2006; Burges et al., 2015; Xian et al., 2015). Pan and Yu (2011) reported that HMs (Cd or/and Pb) reduce the activity of soil enzymes such as acid phosphatase, dehydrogenase, and urease, as well as the soil microbial community. Some researchers investigated the combined impact of HMs and soil characteristics on soil functions and concluded that arylsulfatase is the most sensitive soil enzyme that might be utilized as a marker of soil toxicity (Xian et al., 2015).

### Heavy Metals Responses in Plant System

Heavy metal contamination is a modern ecological issue that pollutes air, water, and soil. This not only results in significant crop yield losses but also raises health risks. In order to mitigate the damage caused by HM contamination, the antioxidative machinery of plants gets triggered.

#### Oxidative Stress and Reactive Oxygen Species

“Reactive oxygen species (ROS)” are reactive chemical species produced from molecular oxygen. Various diverse ROS are present momentarily among all aerobes which include: (a) oxygen-derived non-radicals viz. singlet oxygen (^1/2^O_2_), organic hydroperoxide (ROOH), hydrogen peroxide (H_2_O_2_); and (b) oxygen-derived free radicals viz. superoxide anion (O_2_^–^), alkoxyl (RO⋅) radicals, peroxyl (RO_2_⋅), and hydroxyl (HO⋅) (Circu and Aw, 2010; Shahid et al., 2014; Tamás et al., 2017). Plants tend to produce more ROS, after exposure to HMs as they can disrupt the electron transport chain of the mitochondrial and chloroplast membrane. The increased load of ROS interrupts the redox balance of the cell by causing plasma membrane damage and ion leakage (Dingjan et al., 2016; Anjum et al., 2017), lipid peroxidation, and the disintegration of cellular macromolecules (Carrasco-Gil et al., 2012; Chen et al., 2012; Venkatachalam et al., 2017). The increased amount of Cr in two maize genotypes reduced the content of soluble protein and elevated the level of phenol, proline, and other soluble sugars (Anjum et al., 2017).

#### Genotoxicity

The pathways regulating metal-induced genotoxicity are intricate and are understudied (Cuypers et al., 2011). However, it is clear that HM-induced genotoxicity/DNA damage happens indirectly via the generation of ROS during oxidative stress (Barbosa et al., 2010; Shahid et al., 2014; Aslam et al., 2017). HM-induced nucleic acid impairments have previously been identified in plants such as *Helianthus annuus* (Chakravarty and Srivastava, 1992), *Vicia faba* (Pourrut et al., 2011; Arya et al., 2013; Arya and Mukherjee, 2014), *Solanum tuberosum*, and *Nicotiana tabacum* (Gichner et al., 2006), and *Allium cepa* (Arya et al., 2013; Arya and Mukherjee, 2014; Qin et al., 2015). The oxidation state of HM, its amount, and duration of exposure greatly affects the genotoxic response of any plant (Aslam et al., 2017). The extremely reactive species among ROS is the hydroxyl radical (OH⋅), which can react to and damage all of the DNA molecule’s components (Jones et al., 2011). When ROS react with DNA, it can cause deletion, and modification of nitrogenous bases, breakage of strands, damage to cross-links, and generation of nucleotide dimmers (Gastaldo et al., 2008). When Pb and Cd react with DNA, Yang et al. (1999) detected the formation of 8-hydroxydeoxyguanosine (8-OHdG) adducts, which resulted in the breaking of the strand. Further, Hirata et al. (2011) reported Cr and As-triggered translesion DNA synthesis as a consequence of 8-OHdG production. Several recent studies were made on the genotoxic effects of copper and lead (Qin et al., 2015; Silva et al., 2017; Venkatachalam et al., 2017).

#### Interference With Signaling Pathways

Deregulation of signaling pathways mediated by HM interactions is the main cause behind HM toxicity by influencing G-proteins, growth factor receptors, and receptor tyrosine kinases (Harris and Shi, 2003). HMs also boost H_2_O_2_ production in plants by increasing the synthesis of salicylic acid (SA), jasmonic acid (JA), and ethylene (ET), which interferes with the cell signaling mechanism (Maksymiec, 2007; Schellingen et al., 2014; van de Poel et al., 2015). Plants exposed to As, have higher levels of JA, which stimulates the expression of several signaling and stress-response genes such as MAPK, CDC25, and genes regulating glutathione metabolism (Thapa et al., 2012; Islam et al., 2015).

#### Physiological and Biochemical Response

The anti-oxidative enzymatic machinery of plants such as ascorbate peroxidase (APX), catalase (CAT), glutathione (GSH), glutathione reductase (GR), guaiacol peroxidase (GPX), peroxidase (POX), and superoxide dismutase (SOD) plays a critical role in neutralizing extra ROS (Zhang and Klessig, 2001; Venkatachalam et al., 2017). Increased MDA generation due to enhanced ROS in the cell was observed when two mangrove plants were exposed to HMs (Zhang and Gu, 2007). Similarly, superoxide dismutase (SOD) level was dramatically increased in leaves and roots at low metal concentrations, but dropped drastically at greater concentrations, suggesting a reduction in SOD scavenging ability. *Abelmoschus esculentus* plant grown on sewage sludge reflected an initial increase in chlorophyll content but fall sharply in later stages. The apparent decrease in chlorophyll content might be due to the accumulation of HMs in plants at later stages (Singh and Agrawal, 2008). Similar findings were made in *Vigna radiata* (Singh and Agrawal, 2010) and *Oryza sativa* (Singh and Agrawal, 2010).

## Microbial Resistance to Heavy Metals and Their Mechanisms

**FIGURE 2:**
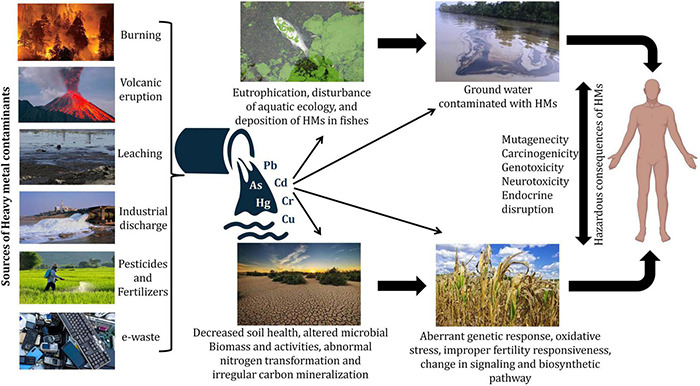
Microbe-mediated environmental remediation of heavy metals.

During stress situations developed by HMs, microorganisms either dies of the toxicity caused by the metal or thrive the situation by evolving mechanism of resistance against metals. For the selection of potent bioremediation agents, microorganisms should develop the mechanism of resistance against the toxicity of metals. Different resistance mechanisms developed by microorganisms like Extracellular barriers, extracellular and intracellular sequestration, active transport of metal ions, and enzymatic detoxification are discussed below (Figure 2). Barriers like cell walls, plasma membrane, and other structures present at the surface like EPS, biofilms restrict the passage of HMs into the cells of bacteria. Microbes’ cell surfaces have a variety of characteristics that prevent metal ions from entering by adsorbing them on their surface and functioning as barriers. E.g., a study by Kumar et al. (2014) showed that isolates of fungi and bacteria can cause biosorption of HMs like copper, lead, and chromium. Tolerance against numerous HMs like copper, zinc, iron, nickel, lead, and cadmium was shown by *Cellulosimicrobium* sp. Chemisorption sites were involved in this resistance mechanism (Bhati et al., 2019). The Biofilms produced by microbes are made up of extracellular polymers that are capable of accumulating metal ions and therefore protect the cells present inside them. The tolerance against lead, zinc, and copper ions has been displayed by the biofilm of *Pseudomonas aeruginosa* (Teitzel and Parsek, 2003). The efficiency to eliminate metal was enhanced from 91.71 to 95.35% by the biofilm of *Rhodotorula mucilaginosa* (Grujić et al., 2017). In addition to biofilms and cell walls, EPS has also been shown to be a barrier against metals, e.g., Adsorption of lead ions was reported in *P. aeruginosa*, *Acinetobacter junii* L. Pb1, and *Azotobacter chroococcum* XU1 (Bramhachari et al., 2007; Rasulov et al., 2013; Kushwaha et al., 2017).

Numerous proteins and metabolic products are found in the cell membrane of the microbes that are capable of making complex structures (chelation) with the metal ions. Extracellular sequestration can be defined as the complexation of metal ions as insoluble compounds or metal ions accumulation by the components of the cell in the periplasm. Copper-inducible proteins CopA, CopB (periplasmic proteins), and CopC (outer membrane protein) are produced by copper-resistant *Pseudomonas syringae* strains that are responsible for the binding of microbial colonies and copper ions. Zinc ions can pass from the cytoplasm and get accumulated into the periplasm of the Synechocystis PCC 6803 strain via the efflux mechanism (Thelwell et al., 1998). Hazardous metals can be reduced by iron and sulfur-reducing bacteria like *Desulfuromonas* spp. and *Geobacter* spp. into less or non-hazardous metals. An obligate anaerobe, *G. metallireducens*, can reduce manganese (Mn) from poisonous Mn (IV) to Mn (II) and uranium (U) from toxic U(VI) to U(II) (IV) (Gavrilescu, 2004). In intracellular sequestration, metal ions are complexed by distinct compounds in the cell cytoplasm. The interaction of metals with the ligands presents in the surface, followed by sluggish transport into the cell, can result in a high concentration of metals within the cells of microorganisms. The ability to accumulate metals intracellularly by bacterial cells has been used in a variety of applications, most notably in waste treatment. With the help of low molecular weight proteins that were rich in cysteine, a cadmium-tolerant *Pseudomonas putida* strain was able to sequester copper, cadmium, and zinc ions intracellularly (Higham et al., 1986). In *Rhizobium leguminosarum* cells, glutathione was also found to be involved in the sequestration of cadmium ions intracellularly (Lima et al., 2006). Lipids, chitin, mineral ions, nitrogen-bearing polysaccharide, polyphosphates, and proteins make up the firm cell wall of fungi. The accumulation of metals by numerous fungi into their spores and mycelium helps in decontaminating metal ions by energy uptake, intracellular and extracellular precipitation, and valence exchange. Another strategy to protect against HM stress is to transport HM ions out from the intracellular environment, which can happen through efflux mechanisms that can effectively regulate intracellular HM ion concentrations (Remenar et al., 2018). Efflux systems have been discovered in a variety of microbes, particularly those isolated from metal contaminated surroundings. Metal exporting proteins, such as ABC transporters, P-type efflux ATPase, cation diffusion facilitator, and proton-cation antiporters are widely distributed in the cell membrane to achieve HM ion efflux. For the export of Cu (II), Cd (II), and Zn (II), Gram-positive bacteria utilize P-type efflux ATPase. With the help of ATPase, an exporting protein on the cell membrane regulates arsenite outflow (Yang et al., 2012; Soto et al., 2019). ABC transporters which also called traffic ATPases can assist microorganisms to survive the stress caused due to HMs by mediating membrane translocation of HM ions (Al-Gheethi et al., 2015; Lerebours et al., 2016; Zammit et al., 2016).

The resistance to HMs ions in microbes is also contributed by the enzymes that biologically transform or chemically modify the HM ions from highly hazardous form to less toxic form (Liu et al., 2017). HM ions’ toxicity can be effectively reduced by changing their redox state via reduction or oxidation reactions (Giovanella et al., 2016). Detoxification enzymes can influence this defensive mechanism, which is also controlled by microbe resistance genes. Through mercuric ion reductase, bacteria like *Bacillus* sp. display resistance to mercury ions (Noroozi et al., 2017). Mercuric reductase transforms the mercuric ion into metallic mercury, which is then discharged into the environment via the cell membrane (Zhang et al., 2012). To reduce toxicity, bacteria such as *Micrococcus* sp. and *Acinetobacter* sp. can oxidize hazardous as (III) into less soluble and non-toxic As (V) (Nagvenkar and Ramaiah, 2010).

## Microbial Mechanism Involved in Heavy Metal Bioremediation

For the elimination of HMs from the polluted sites, bioremediation methods are employed (Pratush et al., 2018). Usually, these methods involve the absorption/adsorption of toxic metallic ions, and this alleviates the related side effects (Njoku et al., 2020). Different natural resources like wood bark/dust, coconut husk/shells, agro wastes, microorganisms, seaweeds, seeds, discarded coffee beans, and aquatic plants, etc. are being used constantly to reduce the number of HM ions from the place of their origin, in which microbes (algae, fungi, bacteria, yeasts, etc.) play a considerable role (Mudila et al., 2019). Microorganisms change the HMs’ ionic state that influences the solubility, bioavailability, and movement in the soil as well as in the aquatic surroundings (Ayangbenro and Babalola, 2017). Mobilization or immobilization of HMs aids microbial remediation, which is then proceeded by oxidation-reduction, chelation, modification of the metallic complex, and biomethylation (Pratush et al., 2018). The enzymatic catalysis by microbes solubilizes the metals with higher oxidation state to lower oxidation state, for instance, *Thiobacillus ferrooxidans* and *T*. *thiooxidans* are responsible for the enzymatic oxidation of Uranium (Cumberland et al., 2016). The isolation of microorganisms responsible for the degradation of HMs could occur from aerobic as well as anaerobic locations. However, in comparison to anaerobic microorganisms, aerobic microbes are more willing for bioremediation (Azubuike et al., 2016). Microorganisms carry out the transportation of HMs utilizing membrane-linked transport mechanisms and transform them into non-hazardous forms (Igiri et al., 2018). Microorganisms use processes like biosorption, bioaccumulation, biotransformation, and bioleaching to stay alive in a metal-polluted environment (Figure 2 and Table 2). These techniques have been used in clean-up processes (Gadd, 2000; Lin and Lin, 2005).

**TABLE 2:** Microbe-mediated remediation and resistance mechanism of heavy metals.

Microbial group	HM contamination	Microorganism	Microbial/Resistance mechanism	References
Bacteria	Cadmium	*Pseudomonas aeruginosa*	Biosorption	Chellaiah, 2018
	Lead	*Bacillus subtilis* X3	Bioimmobilization	Qiao et al., 2019
	Cadmium and lead	*Pseudomonas aeruginosa* and *Bacillus cereus*	Bioaugmentation	Nath et al., 2018
	Cadmium	*Cupriavidus* sp. strain Cd^+2^	Bioprecipitation	Li et al., 2019
	Nickel	*Bacillus* sp. KL1	Biosorption	Taran et al., 2019
	Copper, cadmium, and zinc	*Desulfovibrio desulfuricans*	Extracellular sequestration	Yue et al., 2015
	Copper, palladium, and zinc	*Pseudomonas aeruginosa*		Teitzel and Parsek, 2003
	Cadmium and zinc	*Synechococcus* sp.	Intracellular sequestration	Blindauer et al., 2008
	Mercury, cadmium, and zinc	*Escherichia coli*	Active export	Lerebours et al., 2016
	Mercury	*Bacillus firmus*	Enzymatic detoxification	Noroozi et al., 2017
Algae	Cadmium, zinc, lead, and nickel	*Asparagopsis armata*	Biosorption	Romera et al., 2007
	Ar(V)	*Lessonia nigrescens*		Hansen et al., 2006
	Lead, nickel, and cadmium	*Cystoseira barbata*		Yalçın et al., 2012
	Lead, nickel, cadmium, and zinc	*Codium vermilara*		Romera et al., 2007
Fungi	Copper, lead, and Cr(VI)	*Aspergillus niger*		Dursun et al., 2003
	Lead	*Botrytis cinereal*		Akar et al., 2005
	Copper	*Rhizopus oryzae*		Fu et al., 2012
	Silver	*Pleurotus platypus*		Das et al., 2010

### Bioaccumulation and Biosorption

Both bioaccumulation and biosorption are the processes utilizing which microbes or biomass gets bound to the HMs and pollutants from the surroundings and concentrates them (Joutey et al., 2015). However, the working manner of the processes differs. Biosorption is a process in which microorganisms use their cellular structure to capture HM ions, which they then sorb onto the cell wall’s binding sites (Malik, 2004). This is a passive uptake process and does not depend upon the metabolic cycle. Two common methods for the bioremediation of HMs are adsorption and absorption onto the cell surface of microbes. Adsorption differs from absorption in that it involves the dissolution or permeation of a fluid (the absorbate) by a liquid or solid (the absorbent) (Jovancicevic et al., 1986). Adsorption, on the other hand, is a surface occurrence, whereas absorption affects the full volume of the substance. Various living creatures have been shown to be possible bio sorbents e.g., bacteria like *Magnetospirillum gryphiswaldense, Bacillus subtilis*, algae-like marine microalgae, and *Chaetomorphalinum*, fungi like *Rhizopus arrhizus* and yeast-like *Saccharomyces cerevisiae* (Romera et al., 2006; Wang and Chen, 2009; Zhou et al., 2012). Bacteria, on the other hand, are regarded as the most exceptional biosorbents among all other creatures due to their high surface-to-volume ratios and numerous chemosorption active sites in their cell wall, such as teichoic acid (Beveridge, 1989). Dead bacterial strains have also been considered as promising biosorbents, with biosorption abilities that exceed those of living cells of the same strain. 13–20% increased capacity for the biosorption of chromium ions was shown by dead *Bacillus sphaericus* as compared to the cell of its living strain (Velásquez and Dussan, 2009). However, unlike biosorption, bioaccumulation by microbes is metabolically active and relies upon the import-storage system. In this system, HM ions are transported through the lipid bilayer of the cell membrane into the intracellular spaces or cytoplasm with the help of transporter proteins. This is known as active uptake or bioaccumulation. Endocytosis, ion channels, carrier-mediated transport, complex permeation, and lipid permeation are all involved in HM bioaccumulation in the bacterial membrane (Ahemad, 2012; Geva et al., 2016; Shahpiri and Mohammadzadeh, 2018). Bioaccumulation studies of various metals like lead, nickel, silver, mercury, and cadmium have been reported by Ahemad (2012). The study of cadmium by Rani and Goel (2009) discovered periplasmic and intracellular metal accumulation by *P. putida* 62 BN, and it was performed using transmission electron microscopy. The growing cells of *Bacillus cereus* M116 were shown to accumulate about 20% nickel (II) intracellularly, as reported by Naskar et al. (2020). Lead and chromium accumulation by *Aspergillus niger* and *Monodictys pelagic* was reported by Sher and Rehman (2019). In the process of bioleaching, metal oxides and sulfides from ores deposits and secondary wastes are solubilized by different microorganisms like fungi and bacteria (Mishra et al., 2005; Jafari et al., 2019). Following solubilization, purification is achieved with the help of appropriate methods like ion exchange, selective precipitation, adsorption, and membrane separation (Rohwerder et al., 2003).

### Bioleaching

Bioleaching is performed by an extensive range of microbes and among them, acidophiles are the prominent ones. Acidophiles are chemolithotrophs that oxidize Fe (II) to Fe (III) and/or reduce sulfur to sulfuric acid and flourish in low pH environments, particularly 2.0 or below. Sulfuric acid produces ferric ions and protons, which solubilize metal sulfides and oxides from ores (Srichandan et al., 2014), aiding extraction of metal by segregating metals in the solid phase from the more water-soluble phase. Bioleaching, which uses microorganisms as reduction agents, can also be used to extract and recover heavy metals (Wang and Zhao, 2009). The ability of microorganisms to convert the solid chemical within contaminated soil into a soluble substance that can be removed and recovered determines the efficacy of the recovery process. Due to metal resources being non–renewable, recovering metal from industrial waste water may be a viable option for ensuring heavy metal supply (Atkinson et al., 1998). Bioremediation has been offered by a number of researchers as a way to recover raw materials from effluent (Pollmann et al., 2006; Gadd, 2010). Using an *Annona squamosa*-based absorbent with 0.1 M HCl, Cd(II) recovery up to 98.7% was achieved (Isaac and Sivakumar, 2013). Using *Pseudomonas aeruginosa* biomass with 0.1 M HCl, a Cd(II) recovery up to 82% was achieved. Using volcanic rock matrix-immobilized *P. putida* cells with surface-displayed cyanobacterial metallothioneins at pH 2.35 (Ni et al., 2012), 100% Cu(II) recovery was reported. Using activated sludge at pH 1.0 resulted in a 100% recovery of Cu(II) (Hammaini et al., 2007). The introduction of an indigenous strain *Enterobacter* sp. *J1* resulted in a Cu and Pb recovery of over 90% at pH 2 (Lu et al., 2006).

### Biotransformation

Biotransformation is a process in which structurally a chemical compound is altered, thereby relatively a more polar molecular is synthesized (Asha and Vidyavathi, 2009; Pervaiz et al., 2013). In other words, this contact of metal and microorganisms causes toxic metals and organic compounds to get altered to a comparatively less hazardous form. The development of this mechanism in the microorganisms causes them to acclimatize the environmental changes. Microbial transformations can be attained through the production of new carbon bonds, isomerization, introducing functional groups, oxidation, reduction, condensation, hydrolysis, methylation, and demethylation. Transformation of metals by application of microbes has been reported. *Micrococcus* sp. and *Acinetobacter* sp. oxidize hazardous As (III) into less soluble and non-toxic As(III) and reduce its toxicity (Nagvenkar and Ramaiah, 2010). Thatoi et al. (2014) reported that Cr (VI)-tolerant *Bacillus* sp. SFC 500-1E through NADH-dependent reductase has been shown to lessen the hazardous Cr (VI) to less toxic Cr (III).

## Influence of Environmental Change on the Remediation of Heavy Metal Contaminants

The pH is important for microbial biosorption, and the optimal pH varies depending on the microbe. Firstly, pH influences the enzymatic activity in bacteria, altering the rate of HM microbial metabolism (Morton-Bermea et al., 2002). Secondly, pH alters the microorganism’s surface charge, affecting its ability to adsorb HM ions (Galiulin and Galiulina, 2008). Besides this, pH has an impact on the hydration and movement of a variety of metal ions in the soil (Dermont et al., 2008). Both Rodríguez-Tirado et al. (2012) and Wierzba (2015) found that the rate of HMs removal by microbe upsurges with an increase in pH across a certain range, but after the pH climbs to a specific level, the elimination rate begins to decline. According to a study the ideal pH range for most bacteria, is 5.5–6.5, however there are exceptions (Wang et al., 2001). For instance, *Bacillus jeotgali* thrives at a pH of 7. This could be because as the pH rises over a certain point, some metal ions form hydroxide precipitates, which are less prone to microbial adsorption (Hu et al., 2010; Rodríguez-Tirado et al., 2012). Furthermore, the optimal pH for aerobic microbes and anaerobic microorganisms may differ.

The rate of absorption of HM is mostly influenced by ambient temperature, which impacts the growth and multiplication of microorganisms (Fang et al., 2011). Various bacteria have different optimal temperatures (Acar and Malkoc, 2004) for example, *Acidianus brierleyi* and *Sulfolobussolfa-tataricus* are very thermophilic bacteria, while, *Thiobacillus acidophilus*, *Thiobacillus tepidarius*, and *Thiobacillus ferrooxidans* are medium temperature bacteria.

When it comes to understanding substrate species, there are three things to keep in mind: HM ions, soil additives, and the type of soil. HM adsorption characteristics on different soils might be quite varied. According to a study, beach tidal soil (Freundlich adsorption constant *K* = 93.79) has a larger adsorption capacity than black soil (*K* = 16.41), which is higher than yellow mud (*K* = 1.17), and that the mean desorption rate of soil is Lithic Ochri-Aquic Cambosols in ascending order (0.67%), Fe-accumulic Gleyic Stagnic Anthrosols (3.62%), and Endogleyic Fe-accumulic Stagnic Anthrosols (35.85%) (Chen et al., 1997). Clearly, soil’s adsorption rate and retention of HM ions result in poor mobility of HM ion, making microbial adsorption difficult to achieve (Hu et al., 2010). HM ion species influence HM elimination by changing microbial generation time. The generation period of *Thiobacillus ferrooxidans* on sulfur as a substrate is about 10–25 h, which is significantly longer than the 6.5–15 h generation time on Fe. Moreover, the existence of metal ions in the soil affects microbial enrichment (Kapoor and Viraraghavan, 1997). Individual bioavailability of Pb2+, Cd2+, and Zn2+ in the soil is often more than that of several metal ions, according to Park et al. (2016). The adsorption of Cd2+ alone is 11.2 mg/g. Its adsorption is reduced to 3.15 mg/g in the presence of Zn2+ and Pb2+, with similar results for Zn2+ and Pb2+, displaying the reduction from 19.5 and 2.25 to 8.08 and 0.915 mg/g, respectively. Soil additions can considerably boost microbial removal of HMs, and the concentration of additives can have varied impacts on HM ion leaching rates. Tyagi et al. (2014) found that adding 20 g/L FeSO4.7H2O to a solution increased the leaching rate of Zn and Cu by 2 and 1.9 times, respectively, but not when the concentration was larger than 20 g/L. The adsorption rate of microorganisms is also affected by the concentration of HM ions. To estimate the quantification of accumulative properties of a bio-sorbent, adequate assessment is required in general (Cervantes et al., 2001). The Langmuir model, whose parameters are interpretable and primarily explains the adsorption of a single-layer surface, is one of the most commonly used equations to describe the features and another Freundlich model, is mostly employed to the adsorption equilibrium of the adsorption surface equation (Febrianto et al., 2009). Despite the fact that the Freundlich model is simpler, it grows unbounded, hence the Langmuir model has been more extensively employed than the Freundlich model until recently. The Langmuir model was utilized by some researchers to investigate the influence of HM concentration (Ehrlich, 1997; Brunetti et al., 2012). They discovered that depending on the microorganisms and HM ions investigated, the concentrations of HM ions with the highest adsorption rates change. Though, the trend, which is consistent across all examples, implies that adsorption increases to a certain point and then remains constant as HM ion concentrations rise.

## Modern Approaches in Microbe-Intervened Biotechnologies

### Rhizoremediation: The Phyto-Microbial Remediation System

Rhizoremediation combines two methods for cleansing polluted substrates: phytoremediation and bioaugmentation. Rhizoremediation is the process of using microorganisms found in the rhizosphere of plants that are involved in the phytoremediation process. Application of plants and plant growth-promoting bacteria (PGPB) is being assessed as an effective and environmentally acceptable way for soil renewal and HMs elimination, among the several integrated techniques (Ali et al., 2013; Sati et al., 2022). Many microorganisms in the rhizosphere, such as mycorrhizal fungi and other rhizospheric organisms, can help plants absorb or adsorb HMs (Bojórquez et al., 2016). Joner and Leyval (1997) found that mycorrhizal plants uptake 90, 127, and 131% more Cd than non-mycorrhizal plants, when the concentration of Cd^2+^ in the soil is 1, 10, and 100 mg/kg, respectively. Bissonnette et al. (2010) displayed that mycorrhiza inoculation improves the ability to absorb Cu2+, Cd2+, and Zn2+. Mycorrhizal fungi possess mycelia that grow into the soil, thereby increasing the surface area of plant roots (Trellu et al., 2016). For metal extraction with plants, PGPR including *Azospirillum, Alcaligenes, Agrobacterium, Arthrobacter, Burkholderia, Bacillus, Pseudomonas, Rhizobium*, and *Serratia*, are commonly utilized (Carlot et al., 2002; Glick, 2003). Metal transformation, immobilization, chelation, or solubilization is aided by the production of exopolysaccharides by PGPB, like oxidases, reductases, siderophores, and organic acids which promotes phytoremediation of HMs. PGPB reduces the pH of the soil by producing organic acids, which aids in the removal of HM ions. Metal resistant siderophore-producing bacteria found near the rhizosphere supply nutrients to the plants namely iron, perhaps reducing the negative consequences of metal contamination (Dimkpa et al., 2008; Sinha and Mukherjee, 2008). Siderophore is also responsible for the formation of stable complexes with radionuclides and metals concerning environment like Cd, Ga, Al, Cu, Zn, In, and Pb (Neubauer et al., 2000; Rajkumar et al., 2010). The synergistic effects of bioaugmentation and phytoremediation leading to rhizoremediation may overcome the difficulties that arise when both processes are employed distinctly. Moreover, the remediation of HM with the help of higher plants has also been reported. Wang et al. (2021) also found that planting Salix in Cd-polluted soil improved the diversity of beneficial microbes, such as the bacteria genera *Arthrobacter* and *Bacillus. Anaeromyxobacter, Novosphingobium, Niabella, Niastella, Flavobacterium, Thermomonas, Lysobacter, Pedomicrobium, Solitalea, Devosia, Flavisolibacter, Mesorhizobium, Nitrospira, Rmlibacter*, and *Rubrivivax. Phyllobacterium* and mycorrhizal genera of fungi include *Amanita, Cryptococcus, Conocytes, Actinomucor, Ramicandelaber, Spizellomyces, Xylaria, Rhodotorula, Umbilicaria, Sporobolomyces, Tilletiopsis, Claroideoglomus*, and *Cirrenaliain* plant rhizosphere.

### Genetically Engineered Organisms and Modern Molecular Biology

Bioremediation using microorganisms can degrade and dissipate chemicals of complex substances, making it a long-term solution for reducing HMs contamination in soil (Mosa et al., 2016; Bhatt et al., 2020a,b). Recent advances in genetic engineering, as well as the adequacy of genetically engineered microorganisms/biocatalysts for the restoration of the environment, have shown that they are more capable than natural microbes, particularly for the removal of persistent compounds under natural environments (de Lorenzo, 2009; Bhatt et al., 2020c). By the application of various genetic and metabolic engineering approaches, the genetic material of microbes is modified, and engineered microorganisms are produced which are more efficient thus resulting in enhanced bioremediation. Single-gene editing, pathway construction, and change of existing gene sequences i.e., both coding as well as controlling sequences are included in the aspects of engineering, with a focus on the modification of rate-limiting stages of the metabolic processes (Diep et al., 2018). HMs such as Fe, Cd, As, Cu, Hg, and Ni can now be eliminated with the help of engineered bacteria (D’Souza, 2001; Verma and Singh, 2005; Azad et al., 2014). The rate of degradation, on the other hand, is determined by the catalytic efficiency of enzymes present in the cells or those stimulated to a specific substrate (Kang, 2014). Using recombinant DNA technology, foreign genes from another creature of the same or other species are put into the genome of genetically engineered microorganisms (GEMs). The utilization of genetically modified *Pseudomonas putida* and *Escherichia coli* strain M109 harboring the merA gene to successfully remove Hg from polluted soils and sediments has been reported (Chen and Wilson, 1997; Barkay et al., 2003; Deckwer et al., 2004). Azad et al. (2014) provided a thorough evaluation of the application of genetically modified bacteria and plants in the bioremediation of HMs and other organic pollutant-contaminated environments. According to a study, the addition of the mer operon from *Escherichia coli*, which codes for the reduction of Hg2+, into the genetically modified bacterium *Deinococcus geothemalis* provides the ability to microorganism to lessen the Hg pollution at high temperatures by mer genes (Dixit et al., 2015). *Cupriavidus metallidurans* strain MSR33, which was genetically engineered with a pTP6 plasmid that provided genes merB and merG which regulate the biodegradation of Hg as well as the production of merB and merA, i.e., organomercurial lyase protein and mercuric reductase, was able to reduce Hg contamination from polluted sites (Rojas et al., 2011; Dixit et al., 2015). The insertion of novel genes into *Pseudomonas* cultures using the pMR68 plasmid has also resulted in Hg resistance (Sone et al., 2013). Specific genes in n-alkane-degrading microbes, such as alkB, alkB1, alkB2, alkM, aromatic hydrocarbons: xylE, and polycyclic aromatic hydrocarbons: nidA, ndoB, are frequently found on plasmids that allow horizontal gene transfer and are employed as markers to identify microbial biodegradation (Wolejko et al., 2016). Microbial membrane transporters can be genetically modified to improve the bioremediation of HMs in the environment. Transporters and binding mechanisms play crucial roles in this context of HMs remediation (Manoj et al., 2020). Channels, secondary carriers, and primary active transporters are the three principal types of transporters that are usually emphasized. Their location is in the inner lipid membrane such as Fps, Mer T/P, and GlpF in channel transporters; Hxt7, NixA, and Pho84 in secondary carriers; and cdtB/Ip_3327, MntA, TcHMA3, and CopA in primary active transporters. Some of them, such as the porin channels transporters, may also be found in the outer lipid membrane (Jain et al., 2011). As soon as HMs comes inside the cell, numerous phytochelatins, metallothioneins, and polyphosphates work together for the sequestration of the HMs and changing microorganisms’ HM import-storage systems could boost their ability to extract HMs from water and soil (Diep et al., 2018). Thus, in the fight against harmful compounds in the environment, the use of GEMs to speed up the restoration process is crucial. For the successful implementation of GEMs for bioremediation in adverse environmental conditions, the preservation of recombinant bacterial population in the soil is essential, with appropriate environmental conditions prepared and the recombinant bacteria should be capable to endure antagonism from native bacterial species (Dixit et al., 2015). Consequently, further novel molecular approaches for the screening and isolation of microbes for HM bioremediation should be investigated. Multi-omics comprising genomics, metagenomics, metabolomics, proteomics and transcriptomics, and computational biology techniques have been successfully employed in gene mining that supports system biology research of microorganisms at the genetic level concerning bioremediation of HMs (Subashchandrabose et al., 2018; Pande et al., 2020; Sayqal and Ahmed, 2021). Novel genes implicated in the biodegradation processes of several HM contaminants have been discovered because of high-throughput and next-generation sequencing. New technologies involve gene-editing tools like CRISPR-Cas which possesses the ability to enhance the process of bioremediation by engineering microorganisms with genes engaged in the degradation of, particularly recalcitrant substances. When compared to conventional low-throughput ZFNs and TALENs, CRISPR might be utilized to transmit a preferred set of instructions into the genome of microbe in a straightforward manner because it is a programmable, next-generation approach for high-throughput genetic manipulation (Miglani, 2017). A CRISPR segment is likely for the bioremediation by the application of gRNA-guided dCas9 to control the expression of a gene. As a result, fusing transcription factors with dCas9 can either suppress boost or suppress RNA polymerase transcription, which can cause either upregulation or downregulation of the gene expression or a set of genes of interest. Although CRISPER-based approaches can be employed on a variety of mycobacteria and fungi, further applied research in the area of microbe-based removal of HMs from the environment is needed (Shapiro et al., 2018). With the help of multi-omics, biotechnology has developed a large number of strains. The following are some examples *Arthrobacter, Chlorella* (Gong et al., 2018), *Stenotrophomonas maltophilia* (Cho et al., 2018), *Rhodococcus wratislaviensis, Mycobacterium* (Gołêbiewski et al., 2014), *Alcaligenes eutrophus, Pseudomonas putida* (Cycon and Piotrowska-Seget, 2016), *Cyanobacterium synechocystis*, *Saccharomyces cerevisiae*, *Populus* sp. (Cai et al., 2019), *Candida pelliculosa* strain S-02, *Streptomyces aureus* strain HPS-0, *Aspergillus niger* (Kumar et al., 2018), *Sphingomonas* sp., and *Pseudomonas putida* strain KT2440 (Liu et al., 2019).

### Nanotechnology in Microbial Bioremediation

With the application of chemical or biological methods, several types of nanoparticles have been effectively produced and studied for bioremediation of HMs, over the last decade (Baragaño et al., 2020). The advantages of nano-biosorbents with an ultrafine arrangement and a great surface area include (1) enhancing chemical activity and capacity of adsorption, (2) boosting surface binding energy, and (3) lowering internal diffusion resistance (Khati et al., 2017). As a result, nano-biosorbents could be used as a replacement for traditional biosorbents (Abdi and Kazemi, 2015; Alviz-Gazitua et al., 2019). Latest advancements in the nanobiosorption model have resulted in a number of sophisticated ways that improve the complete efficiency of a conventional biosorption process while also ensuring its economic viability (Devatha et al., 2018). Different functional groups, such as –NH_2_, –COOH, and –OH, are intrinsically present in nanoparticles, and tailoring the appropriate functional groups by activating physically/chemically or by modifying surface has proven to improve elimination of HMs. Bacterial strains can also produce nanoparticles that can aid in the bioremediation of HMs (Arshad et al., 2019). Nanomaterials are combined with microbes to improve reduction of HMs, which makes them more effective as compared to their independent application. Factors in determining the interaction between nanomaterials and microbes include (1) the chemical properties of the nanomaterial, its particle size, coating characteristics, and shape, (2) the chemical properties of the nanomaterial, along with the shape, size of the particle, and its coating characteristics, (3) method of metabolism, (4) the nanomaterial’s crystalline phase, (5) the extent of contamination and, lastly (6) the resistance of nanomaterials to the hazardous contaminant and the prevalent ecological conditions. Microbial biostimulation, bioaccumulation, and biotransformation activities are enhanced by nanocomposites, which increase absorption, adsorption, and the number of chemical processes for the reduction of HMs (Tan et al., 2018). Microbes are trapped within nanomaterials to create a nanocomposite; for example, immobilization of gram-negative *Halomonas* sp. within polyvinyl pyrrolidone-coated iron oxide nanoparticles was confirmed to eliminate Pb (II) and Cd (II) (Cao et al., 2020). On the other hand, the microbe can function as a nanoparticle synthesizer, a method known as green synthesis. Though separating or recovering HMs from nanomaterials, is a time-consuming/laborious technique and hence magnetic nanoparticles have gained considerable attention in recent years, wherein surface amendment, coating of iron/iron oxides, and encapsulation focused for simple separation or retrieval of HMs.

## Conclusion

Throughout the world, HM contamination causes severe environmental problems. In this review, several technical strategies i.e., microbe-based as well as hybrid have been discussed, that are currently being employed to mitigate HM contamination in soils and other contaminated surroundings. Because of the contribution in the regulation of biogeochemical cycles that influence climate, soil structure, and fertility, the environmental microbiome is thought to play a critical role. Microbe-mediated bioremediation should be given high attention from a practical standpoint since microbes have a variety of natural roles and mechanisms that considers them a great candidate for the clean-up of the polluted site, management of wastes, and sustainable agriculture. Although microbes are being employed to improve the effectiveness of HM removal from the soil, there is still room for improvement.

## Directions to the Future Research

To create approaches that support better tolerance of HMs in microbes, more emphasis should be placed on understanding the physical, chemical, and biological characteristics of microbes in the prevalence of HMs in soil, water, and gaseous surroundings (Njoku et al., 2020). Furthermore, the application of additives in bioremediation, such as surfactants, might expand the region of the interphase between microorganisms and pollutants, pushing microbes beyond their bioremediation limits. Recently, yeast has been genetically modified to have plant-like properties and to act as hyperaccumulators of several HMs in the aqueous environment (Sun et al., 2020). Other bacteria could be developed in the same way to help in clean-up of HMs. More emphasis should be paid to algae in future study studies, as it is considered an effective microbe for the sorption of HMs from the soil. Because of the better genetic abilities and tolerance to HMs, omics-based techniques are advantageous for the production of improved industrial strains that are tolerant to the prevalent environmental surroundings (Hemmat-Jou et al., 2018). Furthermore, as mentioned in this study, the application of nano- and nano (bio)technologies has enormous ability to promote the use of microbial technologies to deal with HMs pollution. When nanotechnology and microbe-based technology are coupled in environmental restoration procedures, the nanoparticles will promote the elimination of greater pollutant loads, reducing the toxicity-based inhibition of the contaminant on the microbe (Ma and Zhang, 2008). As a result, combining various traditional procedures and current technology could be a potential choice if they could improve relevant material qualities and speed up the restoration process. All life forms and the natural ecosystem are in danger from pollution caused by HMs in soil, water, and agrarian land. Sustainable policies have been developed and revised regularly; nevertheless, awareness of the negative effects, as well as knowledge of how to reduce HMs contaminantion in the soil, should be expanded.

## Author Contributions

VP conceptualized and wrote the manuscript. SP wrote the manuscript and made the diagrams. DS wrote the manuscript. PB and MS supervised, helped in writing, reviewing, and editing the manuscript. All authors contributed to the article and approved the submitted version.

## Conflict of Interest

The authors declare that the research was conducted in the absence of any commercial or financial relationships that could be construed as a potential conflict of interest.

## Publisher’s Note

All claims expressed in this article are solely those of the authors and do not necessarily represent those of their affiliated organizations, or those of the publisher, the editors and the reviewers. Any product that may be evaluated in this article, or claim that may be made by its manufacturer, is not guaranteed or endorsed by the publisher.
